# Common Origin of Filler Network Related Contributions to Reinforcement and Dissipation in Rubber Composites

**DOI:** 10.3390/polym13152534

**Published:** 2021-07-31

**Authors:** Sriharish Malebennur Nagaraja, Sven Henning, Sybill Ilisch, Mario Beiner

**Affiliations:** 1 Fraunhofer Institut für Mikrostruktur von Werkstoffen und Systemen IMWS, Walter-Hülse-Str. 1, 06120 Halle (Saale), Germany; sriharish.malebennur@imws.fraunhofer.de (S.M.N.); sven.henning@imws.fraunhofer.de (S.H.); 2 Trinseo Deutschland GmbH, E 17, 06258 Schkopau, Germany; SIlisch@Trinseo.com

**Keywords:** rubber composites, visco–elastic filler network, dissipation, reinforcement

## Abstract

A comparative study focusing on the visco–elastic properties of two series of carbon black filled composites with natural rubber (NR) and its blends with butadiene rubber (NR-BR) as matrices is reported. Strain sweeps at different temperatures are performed. Filler network-related contributions to reinforcement ($\Delta G^{\prime }$) are quantified by the classical Kraus equation while a modified Kraus equation is used to quantify different contributions to dissipation ($\Delta G_{D}^{\prime \prime }$, $\Delta G_{F}^{\prime \prime }$). Results indicate that the filler network is visco-elastic in nature and that it is causing a major part of the composite dissipation at small and intermediate strain amplitudes. The temperature dependence of filler network-related reinforcement and dissipation contributions is found to depend significantly on the rubber matrix composition. We propose that this is due to differences in the chemical composition of the glassy rubber bridges connecting filler particles since the filler network topology is seemingly not significantly influenced by the rubber matrix for a given filler content. The underlying physical picture explains effects in both dissipation and reinforcement. It predicts that these glassy rubber bridges will soften sequentially at temperatures much higher than the bulk $T_{g}$ of the corresponding rubber. This is hypothetically due to rubber–filler interactions at interfaces resulting in an increased packing density in the glassy rubber related to the reduction of free volume. From a general perspective, this study provides deeper insights towards the molecular origin of reinforcement and dissipation in rubber composites.

## 1. Introduction

Nano-sized filler particles incorporated into a rubber matrix result in materials offering an array of synergistic mechanical properties. Filler particles modify in particular the visco–elastic properties of rubber composites [1,2,3]. Although mechanical properties of rubber composites, specific parameters of nano-sized fillers and filler–matrix interactions have been extensively investigated, a conclusive physical picture describing the mechanisms determining dissipation and reinforcement in rubber composites on the microscopic level is still missing [1,4,5,6,7,8]. A better understanding of these mechanisms is, however, necessary to meet the challenging demands of rubber composites for tire applications. The central task of tire research activities is to solve a multiparameter optimization problem, in particular to optimize in parallel rolling resistance, wet grip and abrasion [9,10,11]. From a more fundamental point of view, this means that one has to design rubber composites fulfilling certain requirements regarding reinforcement and dissipation. These rubber composites are supposed to have low dissipation in the frequency range relevant for rolling resistance, but high dissipation in the frequency range determining the wet grip.

The nature of the filler network is debated already for more than 50 years [4,5]. Various numerical [11,12,13,14] and experimental studies by DMA, DSC, dielectric spectroscopy and NMR [15,16,17,18,19] came to the conclusion that there is a small fraction of immobilized rubber located at the surface of nano-sized filler particles. Immobilized rubber layers in the glassy state are assumed to be formed due to physical adsorption of rubber on the filler surface with a storage modulus that is about 1000 times higher than the bulk rubber. However, the lack of a conclusive methodology to directly detect the immobilized rubber led to different hypotheses, interpretations, and contradicting conclusions [5,20,21,22]. In many studies, layer thicknesses of about 2–3 nm are estimated [5,15,16,23,24], while significantly larger thicknesses are reported in other cases. Other authors even rejected the existence of glassy rubber layers [21]. Systematic studies by frequency-temperature-dependent strain sweeps and solid-state NMR on industrially relevant S-SBR compounds filled with silica particles in the last decade demonstrated the occurrence of a small fraction of immobilized rubber (1–3%) softening sequentially during heating. The results of this systematic study showed clearly that the volume of the glassy rubber fraction is increasing with filler loading and measurement frequency, but decreasing with temperature [25,26].

Various findings suggest that immobilized rubber bridges connecting neighbored nanoparticles are of extraordinary importance for the properties of the filler network in highly filled rubber composites. A conclusion of relevant studies is that the filler network contains not only solid-like filler particles, but also glassy rubber bridges, which will soften in the application relevant frequency–temperature range. Accordingly, the filler network shows visco-elastic properties [25,26]. An analysis of strain sweeps $G^{\prime }$(γ) performed at different temperatures and frequencies by the original Kraus model [27] has been used to demonstrate the importance of glassy rubber bridges for reinforcement. The reduction in the filler network strength $\Delta G^{\prime }$ with increasing temperature and decreasing measurements frequency is understood as a strong argument for the dominant role of glassy rubber bridges in the filler network, [25,28]. It is concluded that glassy rubber bridges are the weakest part of the visco-elastic filler network determining its mechanical strength.

In a previous communication [29], we have highlighted the importance of glassy rubber bridges for the dissipative properties of rubber composites. Two filler network-related contributions to dissipation are quantified based on strain sweep data for the loss modulus $G^{\prime \prime }\left(\gamma \right)$ with the help of an adequately modified Kraus equation. One contribution ($\Delta G_{D}^{\prime \prime }$) is related to dissipation due to periodic deformation of intact glassy rubber bridges, while the second ($\Delta G_{F}^{\prime \prime }$) has to do with fracturing these bridges. Both contributions are dependent on filler fraction, measurement temperature and frequency demonstrating the visco-elastic nature of the filler network in accordance with the physical picture derived from earlier studies focusing on reinforcement. An approach describing main contributions to reinforcement and dissipation based on common physical pictures is, however, so far absent.

The objective of this work was to get deeper insights towards the molecular origin of reinforcement and dissipation in rubber composites. Temperature-dependent trends in dissipation and reinforcement are compared for natural rubber and natural rubber–butadiene rubber blends filled with an identical grade of carbon black. The importance of glassy rubber bridges being part of the viscoelastic filler network for both—reinforcement and dissipation—is demonstrated.

## 2. Experimental

### 2.1. Materials

Rubbers used in this work are a natural rubber (NR) being Standard Vietnamese Rubber 10 (SVR 10) and a high cis 1,4 butadiene rubber (BR) from Trinseo Deutschland GmbH Schkopau with the trade name BUNA${}^{\mathrm{TM}}$ cis 132. The carbon black used is N234 with a BET surface area of about 125 m${}^{2}$/g, vulcanization system contains sulfur along with *N*-cyclohexyl-2-benzothiazylsulfenamide (Rhenogran CBS-80, Rhein Chemie Additives) as accelerators and diphenylguanidine (Rheocure DPG, Rhein Chemie Additives, Pittsburgh, PA, USA), together with 99% pure zinc oxide and 98% pure stearic acid (Carl Roth GmbH, Karlsruhe, Germany) as activators. Both NR and NR-BR blend (ratio 70:30) are filled with varied volume fractions of N234 (4% $\leq \Phi_{CB}\leq$ 21%) corresponding to (10 to 55 phr).

### 2.2. Rubber Processing

Rubber mixing is carried out in two-steps. The first step involves mechanical mixing of rubber and incorporation of carbon black particles into the rubber matrix along with other additives (Zinc oxide, Stearic acid). The mixing machine used was a HAAKE PolyLab${}^{\mathrm{TM}}$ kneader with mixing chamber having a volume of 78 cm${}^{3}$ and Banbury rotors. The processing parameters for the NR and NR-BR composites are identical. The fill factor was 0.68, rotor speed was 75 rpm, the initial temperature was 80 ${}^{\circ }$C and the mixing time was 10 min. The second step was to introduce accelerators and vulcanizing agents. This process was done with a fill factor of 0.66 at temperatures lower than 110 ${}^{\circ }$C for 3 to 5 min. Both the NR and NR-BR composites are vulcanized finally at 150 ${}^{\circ }$C in a hydraulic press for a duration of time corresponding to t95 obtained from MDR (Moving Die rheometer) measurements.

### 2.3. Methods

*Dynamic shear measurements* with variable strain amplitude were done using an Anton Paar MCR501 Twin-Drive rheometer. Rectangular specimen of dimension 2 × 8 × 30 mm${}^{3}$ were stamped out of rubber composite sheets and clamped with approximate length of 20 mm in the rheometer tools. Strain amplitude was increased logarithmically from 0.001 to 40% at a fixed angular frequency (ω) of 10 rad/s. The normal force was maintained close to zero during the measurement. Note that for each measurement temperature, T = 0, 25, 60 ${}^{\circ }$C a new specimen was used. Storage modulus $G^{\prime }\left(\gamma \right)$ and loss modulus $G^{\prime \prime }\left(\gamma \right)$ depending on strain amplitude are used afterward to quantify the different contributions to the reinforcement and dissipation. Each strain sweep was done only once, but the reproducibility was carefully checked based on selected samples.

*Transmission electron microscopy (TEM)* studies on rubber composites were performed by means of a FEI Tecnai G2 TEM operated at 200 kV. Ultra thin sections of a thickness of 60 nm were prepared by cryo-ultramicrotomy using a RMC Power Tome with CRX cryo chamber equipped with a Diatome diamond knife. The software cell${}^{F}$ (Olympus Soft Imaging Solutions GmbH, Münster, Germany) was used for image processing.

## 3. Results

**Figure 1:**
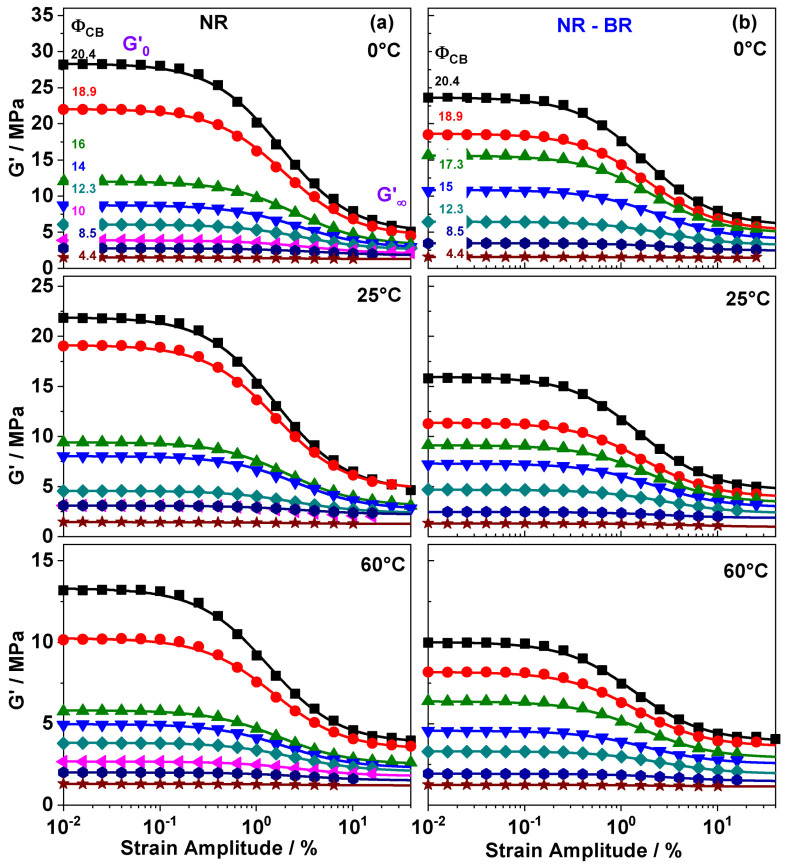
Dynamic storage modulus $G^{\prime }$ as a function of shear strain amplitude γ for (**a**) NR and (**b**) NR-BR composites containing varied fractions of carbon black $\Phi_{CB}$ (volume fractions are labeled in the upper plot). All measurements are done at 10 rad/s and temperatures of 0 ${}^{\circ }$C, 25 ${}^{\circ }$C and 60 ${}^{\circ }$C. The lines are fits based on the Kraus Equation (Equation (1)). The obtained $\Delta G^{\prime }$ values are given in Table 1. Experimental uncertainties of the mechanical measurements are of the order of symbol size.

Storage modulus $G^{\prime }\left(\gamma \right)$ data from dynamic mechanical measurements with strain amplitudes γ in the range from 0.001% to 40% for NR and NR-BR composites containing a varied fraction of carbon black (CB) are shown in Figure 1. The amplitude dependent behavior of $G^{\prime }\left(\gamma \right)$ changes for both investigated series of rubber composites at filler fractions of about $\Phi_{CB}\approx 10$ vol.% qualitatively. For low filler fractions $G^{\prime }\left(\gamma \right)$ is nearly constant over the complete range of strain amplitudes, whereas a sigmoidal decrease is observed in $G^{\prime }\left(\gamma \right)$ for composites containing filler fractions $\Phi_{CB}\geq 10$ vol.%. This phenomenon is known as the Payne effect [4] and is commonly found in composites containing a filler network formed by percolation of filler particles above a certain threshold $\Phi_{C,CB}$ being about 10 vol.% for both investigated rubber composite series. For filler fractions $\Phi_{C,CB}$, the magnitude of $G_{0}^{\prime }$ at small strain amplitudes γ < 1% shows a strong dependence on filler content while $G_{\infty }^{\prime }$ at large strain amplitudes γ > 25% is weakly influenced by $\Phi_{CB}$. The difference $\Delta G^{\prime }=G_{0}^{\prime }-G_{\infty }^{\prime }$ can be attributed to the load carrying capacity of the filler network and grows accordingly with increasing filler contents. Special interest here is the temperature dependence of the load carrying capacity, which is measured at T = 0, 25, 60 ${}^{\circ }$C. The values of $G_{0}^{\prime }$ and $\Delta G^{\prime }$ show a pronounced decrease with increasing temperature. For instance, in highly filled NR composite containing $\Phi_{CB}=20.4$ vol.%, the value of $\Delta G^{\prime }$ is approximately halved when the temperature is increased from 0 to 60 ${}^{\circ }$C. Interestingly, the behavior of CB filled NR-BR composites is qualitatively quite similar to that of corresponding NR composites, but the $G_{0}^{\prime }$ as well as $\Delta G^{\prime }$ values are commonly lower. This is a common finding for all filler fractions above $\Phi_{C,CB}$ and the origin of this feature has been related to the existence of glassy rubber bridges being part of the filler network [25].

In order to quantify the filler network contributions to reinforcement the $G^{\prime }\left(\gamma \right)$ sweeps are approximated using the Kraus Equation [27]

(1) $$G_{\gamma }^{\prime }=\frac{G_{0}^{\prime }-G_{\infty }^{\prime }}{1+{\left(\frac{\gamma }{\gamma_{c}}\right)}^{2m}}+G_{\infty }^{\prime }.$$

The difference $\Delta G^{\prime }=G_{0}^{\prime }-G_{\infty }^{\prime }$ determines the filler network contribution to reinforcement and the exponent *m* (fixed here to 0.6) describes the shape of the sigmoidal decrease in $G^{\prime }\left(\gamma \right)$ that appears near the characteristic strain amplitude $\gamma_{c}$.

**Figure 2:**
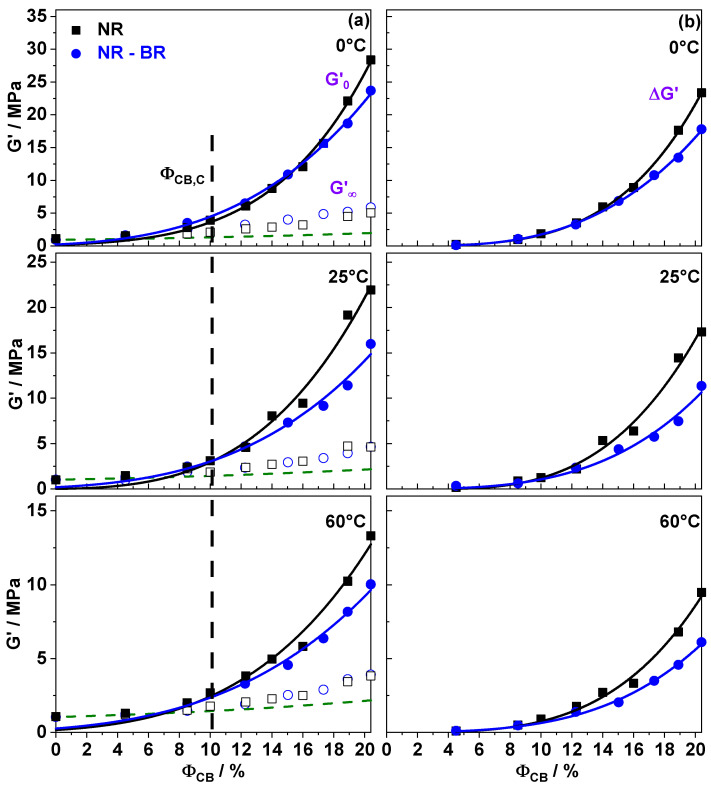
Filler fraction $\Phi_{CB}$ dependent fit parameters for different temperatures. (**a**) $G_{0}^{\prime }$ (filled) and $G_{\infty }^{\prime }$ (open) are Kraus fitting parameters for NR (squares) and NR-BR (circles) composites. Black dotted vertical line represents the percolation threshold $\Phi_{C,CB}$ and green dotted line is hydrodynamic reinforcement predictions according to Guth–Gold [30]. (**b**) Strength of the filler network $\Delta G^{\prime }$= $G_{0}^{\prime }$ − $G_{\infty }^{\prime }$ for NR (squares) and NR-BR (Circles) composites. Lines connecting the points is a guide to the eye.

Kraus fits with m=0.6 approximate the experimental data for all investigated composites and conditions obviously quite well (Figure 1). The parameters taken from fits are presented in Figure 2. As expected, $G_{0}^{\prime }$ and $G_{\infty }^{\prime }$ show a weak dependence for filler fractions below $\Phi_{C,CB}$ 10%, while $G_{0}^{\prime }$ increases drastically above $\Phi_{C,CB}$. This applies to both NR and NR-BR composites and for all investigated temperatures. The percolation threshold ($\Phi_{C,CB}$), however, is similar for both investigated series and not significantly influenced by temperature. For highly filled composites the filler network contributions quantified by $\Delta G^{\prime }$ are significantly larger than those caused by hydrodynamic effects and occluded rubber related to $G_{\infty }^{\prime }$ [30]. The direct comparison in Figure 2b of both investigated series clearly shows that the $\Delta G^{\prime }$ values for NR composites are systematically higher than those of NR-BR composites for $\Phi_{C,CB}$. This finding indicates that filler network contributions to reinforcement are significantly dependent on the rubber matrix. Interestingly, the $G_{\infty }^{\prime }$ values are almost same in both the series at a given temperature and filler fraction, showing that the filler network independent contributions to reinforcement are weakly influenced by the rubber matrix composition.The $G_{\infty }^{\prime }$ values are commonly a bit higher than the Guth–Gold prediction [30] probably due to occluded rubber contributions.

**Figure 3:**
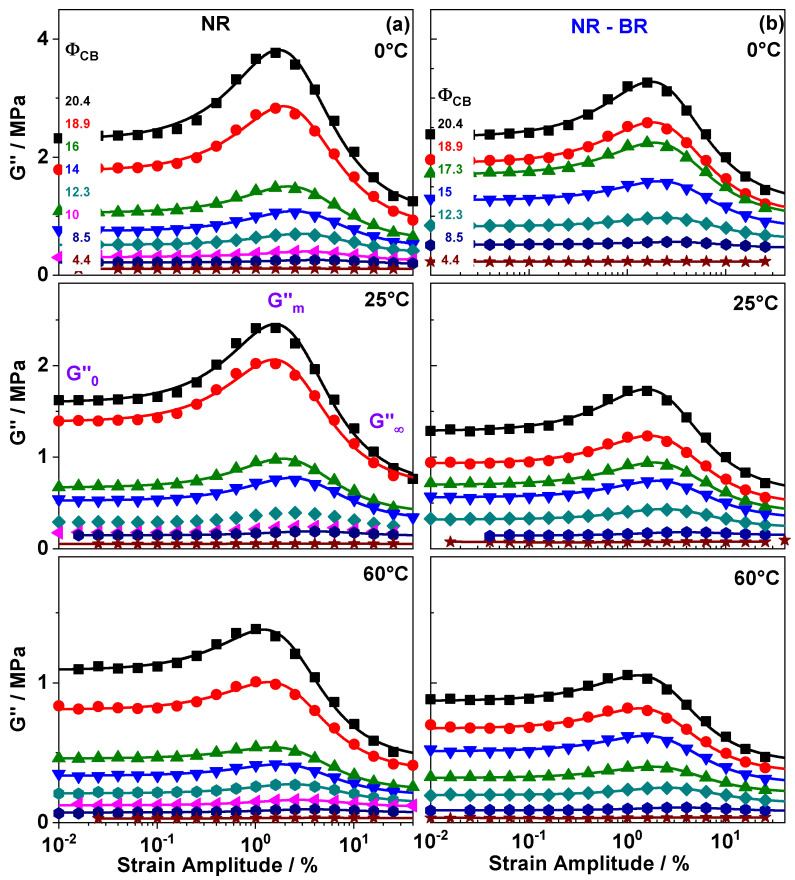
Dynamic loss modulus $G^{\prime \prime }$ as a function of shear strain amplitude γ for (**a**) NR and (**b**) NR-BR composites with varied fraction of carbon black $\Phi_{CB}$ (Volume fractions are labeled in the upper plot). All measurements are done at 10 rad/s and temperatures of 0 ${}^{\circ }$C, 25 ${}^{\circ }$C and 60 ${}^{\circ }$C. The lines over the data points are fits based on the modified Kraus Equation (Equation (2)). The values obtained for $\Delta G_{D}^{\prime \prime }$ and $\Delta G_{F}^{\prime \prime }$ are given in Table 1. Experimental uncertainties of the mechanical measurements are of the order of symbol size.

Figure 3a shows the strain dependence of dissipation, i.e., loss modulus $G^{\prime \prime }\left(\gamma \right)$ for NR composites. As seen in $G^{\prime }\left(\gamma \right)$ for composites containing small amount of filler ($\Phi_{CB}<\Phi_{C,CB}$) practically no dependence on strain amplitude is observed although $G^{\prime \prime }\left(\gamma \right)$ values do increase with $\Phi_{CB}$ slightly. In composites containing large amount of filler ($\Phi_{CB}>\Phi_{C,CB}$), the $G_{0}^{\prime \prime }$ values at small strain amplitudes (γ < 1%) do raise strongly with increasing filler content. The values $G_{\infty }^{\prime \prime }$ at large strain amplitudes (γ > 25%) remain commonly significantly smaller. In addition, a peak is observed in $G^{\prime \prime }\left(\gamma \right)$ at intermediate strain amplitudes that is a typical feature in composites containing percolated filler network. The peak height depends on filler fraction and temperature, as well as composition of the rubber matrix (Figure 3b).

Already Payne [4] and Kraus [27] have related the peak in $G^{\prime \prime }\left(\gamma \right)$ to heat caused by the breaking of the filler network. Accordingly, the dependence on filler fraction has been discussed earlier. The dependence on temperature has not been systematically investigated in the past. Interestingly, an additional sigmoidal contribution to dissipation exists. The dependence of this contribution on strain amplitude (γ) is similar to the shape of $G^{\prime }\left(\gamma \right)$ in the same amplitude range. The sigmoidal contribution to $G^{\prime \prime }\left(\gamma \right)$ is superimposed by the peak and can be quantified by approximating the $G^{\prime \prime }\left(\gamma \right)$ data by a modified Kraus equation having the form

(2) $$G_{\gamma }^{\prime \prime }\;=\;\frac{G_{0}^{\prime \prime }-G_{\infty }^{\prime \prime }}{1\;+\;{\left(\frac{\gamma }{\gamma_{c}}\right)}^{2m}}\;+\;\frac{2\left(G_{m}^{\prime \prime }-G_{\infty }^{\prime \prime }\right){\left(\frac{\gamma }{\gamma_{c}}\right)}^{m}}{1+{\left(\frac{\gamma }{\gamma_{c}}\right)}^{2m}}\;+\;G_{\infty }^{\prime \prime }\;.$$

$G_{0}^{\prime \prime }$ accounts for dissipation at small strain amplitude, while the dissipation at large strain amplitudes is quantified by $G_{\infty }^{\prime \prime }$ and $G_{m}^{\prime \prime }$ identifies the height of the peak. A similar equation was considered among others earlier by Ulmer [31] without any molecular interpretation. Finally, he favored other versions. However, we could show recently that Equation (2) is approximating $G^{\prime \prime }\left(\gamma \right)$ data for rubber composites commonly quite well and that it allows to identify different dissipative contributions together with their molecular origin.

The fit lines in Figure 3 do evidence that Equation (2) also approximates well the experimental $G^{\prime \prime }\left(\gamma \right)$ data for rubber composites with high filler fractions ($\Phi_{CB}\geq \Phi_{C,CB}$) investigated in this work. This applies to all three different temperatures and both matrices, i.e., NR and NR-BR composites. The obtained fit parameters allow us to quantify different contributions to dissipation. In particular, the filler network contribution to $G_{\gamma }^{\prime \prime }$ can be quantified using $\Delta G_{D}^{\prime \prime }$ = $G_{0}^{\prime \prime }$−$G_{\infty }^{\prime }$ as well as $\Delta G_{F}^{\prime \prime }$ = $G_{m}^{\prime \prime }$−$G_{\infty }^{\prime }$. The intensity $\Delta G_{F}^{\prime \prime }$ related to dissipation released due to breaking of the filler network [4,27] is increasing with increasing filler content since the peak height rises. Another common observation is that $\Delta G_{D}^{\prime \prime }$ also raises with filler fraction since the $G_{0}^{\prime \prime }$ values at small strain amplitudes are amplifying with increasing filler content. Measurements done at different temperatures show that with increasing temperature, both contributions, $\Delta G_{F}^{\prime \prime }$ and $\Delta G_{D}^{\prime \prime }$, do decrease systematically.

**Figure 4:**
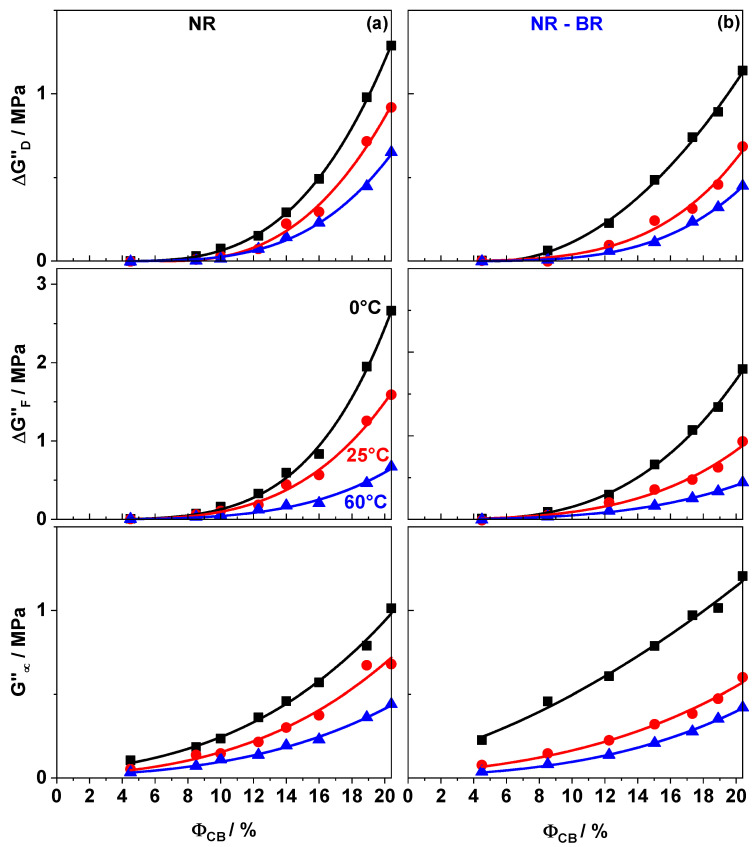
Filler fraction dependence of $\Delta G_{D}^{\prime \prime }$, $\Delta G_{F}^{\prime \prime }$ and $G_{\infty }^{\prime \prime }$ for (**a**) NR and (**b**) NR-BR composites at three different temperatures 0 ${}^{\circ }$C (squares), 25 ${}^{\circ }$C (circles) and 60 ${}^{\circ }$C (triangles). Lines connecting the points is a guide to the eye.

Figure 4 shows the dependencies of all the three contributors to dissipation—the peak height $\Delta G_{F}^{\prime \prime }$, the step height $\Delta G_{D}^{\prime \prime }$, and the value at large amplitudes $G_{\infty }^{\prime \prime }$. The filler network related contributions, $\Delta G_{D}^{\prime \prime }$ and $\Delta G_{F}^{\prime \prime }$, show a strong dependence with filler fraction and temperature. Both decrease with increasing temperature, but increase with filler content. The trend in the non-filler network related contribution $G_{\infty }^{\prime \prime }$ are qualitatively similar, but the changes in the absolute values are much less. Comparing the values of both the filler network related contributions to dissipation in NR and NR-BR composites the influence of matrix composition is again clearly visible. In NR composites $\Delta G_{D}^{\prime \prime }$ and $\Delta G_{F}^{\prime \prime }$ values are higher in comparison to NR-BR composites at a given temperature and filler content. This trend corresponds to that one observed for the filler network contributions to reinforcement ($\Delta G^{\prime }$). An important result of the comparisons made in this study is that $\Delta G^{\prime }$, $\Delta G_{D}^{\prime \prime }$ and $\Delta G_{F}^{\prime \prime }$ show a similar dependence on filler content, temperature and matrix composition.

**Figure 5:**
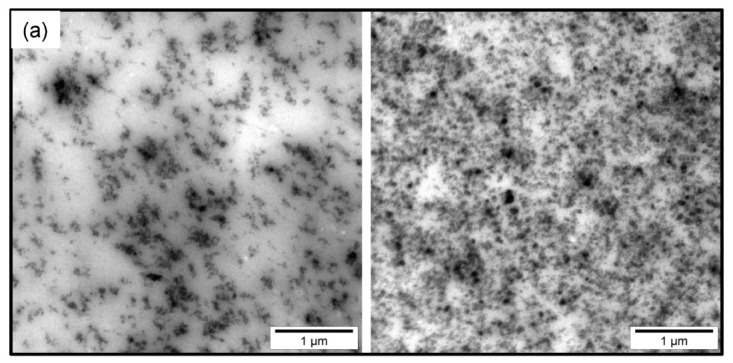

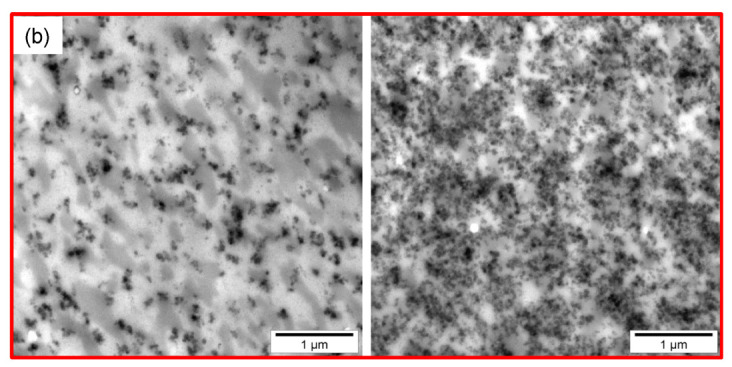
TEM micrograph (**a**) NR composites containing 4.5 and 16 vol.% CB as well as (**b**) NR-BR composites containing 4.5, 15 vol.%. Dark gray domains of BR domains are dispersed in light gray NR matrix.

**Figure 6:**
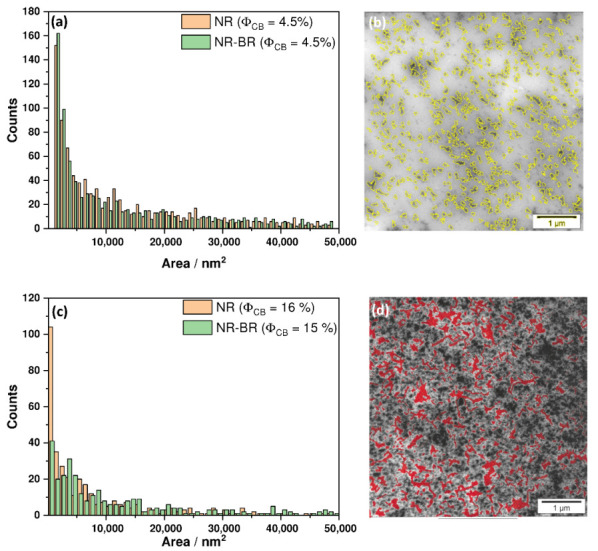
Histograms showing (**a**) the cluster size distribution for NR and NR-BR samples filled with 4.5 vol.% carbon black and the (**c**) size distribution of unfilled areas for NR and NR-BR composites containing $\Phi_{CB}$ 16 or 15 vol.%, respectively. In the TEM images, the identified filler clusters and the unfilled regions are highlighted by color for NR composites containing (**b**) 4.5 vol.% CB and (**d**) 16 vol.% CB, respectively.

An arising question is why there are significant differences between the mechanical properties of rubber composites with NR and NR-BR matrix. In order to contribute to a better understanding of these differences TEM images have been prepared (Figure 5) for representative samples of both rubber composite series with $\Phi_{CB}$ = 4.5 vol.% as well as $\Phi_{CB}\approx$ 15 vol.%. As expected, NR-BR composite show a two-phasic morphology where BR domains with a typical dimensions in the range of a few 100 nm are distributed uniformly in NR matrix. For both samples with lower filler fraction well dispersed filler aggregates are found throughout the matrix, which are isolated from each other. As the filler content is well below the percolation threshold, the level of filler dispersion can be identified via the aggregate size distribution. A comparison of representative histograms of the aggregate sizes for both samples with $\Phi_{CB}$ = 4.5 vol.% is given in Figure 6a. The differences regarding the aggregate size distribution and average aggregate size (0.017268 and 0.016395 μm${}^{2}$ for NR and NR-BR, respectively) is relatively small for a given thickness of the cryomicrotomed sections of about 50 to 60 nm. The shape of the aggregates seems to be also preserved in both single and blend-based rubber composites. Although, the filler fraction $\Phi_{CB}$ = 4.5 vol.% is well below the percolation threshold, it is relatively complicated to quantify the localization of the filler in both rubber components. However, there seem to be clear indications for an over-proportional fraction of filler in the minority BR phase as seen in Figure 5b. A quantification of the filler dispersion in case of composites containing 15–16 vol.% of CB, i.e., amounts clearly above the percolation threshold, is much more complicated since the filler aggregates overlap and are connected to a percolating filler network. Comparative aggregate size distributions cannot be used any longer at such filler fractions. An attempt is made to quantify the filler dispersion in both single and blend composites based on the areas of the unfilled regions in TEM images for 50 to 60 nm thick sections. The unfilled regions are identified and measured by drawing polygons. The procedure is sketched in Figure 6d and the distributions of unfilled area sizes are compared for NR and NR-BR composites. The graph shows that the distribution of unfilled areas in the NR composite is not significantly different from that in the related NR-BR composite. The statistical average sizes obtained for 350 unfilled areas are 0.013931 μm${}^{2}$ for the NR composite and 0.017843 μm${}^{2}$ for the NR-BR composite, respectively. Another parameter that can be compared is the peak position in the histograms, which seems to also not be very different for both composites. This can be interpreted as a clear indication for an almost identical filler network topology above the percolation threshold for NR and NR-BR composites despite significant differences in the rubber matrix composition [32].

## 4. Discussion

### 4.1. About the Common Origin of Filler Network Related Contributions to Reinforcement and Dissipation in Rubber Composites

Temperature-dependent strain sweep measurements on NR and NR-BR composites with variable filler contents ($\Phi_{CB}$) clearly indicate that the strength of the filler network $\Delta G^{\prime }$ is reducing with increasing temperature. This finding is in excellent agreement with findings for S-SBR-silica composites studied earlier and supports the idea that glassy rubber bridges are commonly part of a visco-elastic filler network in rubber composites [5,13,15,25,26]. Following the Kraus model [27]—approximating the experimental data for $G^{\prime }\left(\gamma \right)$ quite well—the filler network strength $\Delta G^{\prime }$ can be approximated based on rate equations considering the number of “contacts between filler particles (or primary aggregates)” $N_{0}$. From our point of view, these “contacts” have to be understood as glassy rubber bridges in specification of the original approach presented by Kraus. As $N_{0}$ increases with increasing filler fraction in the percolated state the strength of filler network related to $\Delta G^{\prime }$ also raises. The decrease of $\Delta G^{\prime }$ with increasing temperature can be related to a sequential softening of the glassy rubber bridges with increasing temperature. Accordingly the number of intact glassy rubber bridges $N_{0}$ and the volume/diameter decreases with temperature. This explains the observed trends in $\Delta G^{\prime }$ for NR as well as NR-BR composites quite well. A common origin of the described finding is, according to this physical picture, the existence of glassy rubber bridges probably formed due to strong physical adsorption of rubber segments on the filler surface. This is speculatively leading to a more dense packing of the rubber layer on the filler surface accompanied by a several 10 K higher glass temperature ($T_{g}$) compared to the bulk-like rubber far away from filler particle surfaces. Note that the existence of such immobilized/glassy rubber layers with a thickness of about 1–2 nm corresponding to volume fractions of about 1–3% of the entire rubber matrix in highly filled rubber composites is also reported in various other studies [5,15,24,25,33].

**Figure 7:**
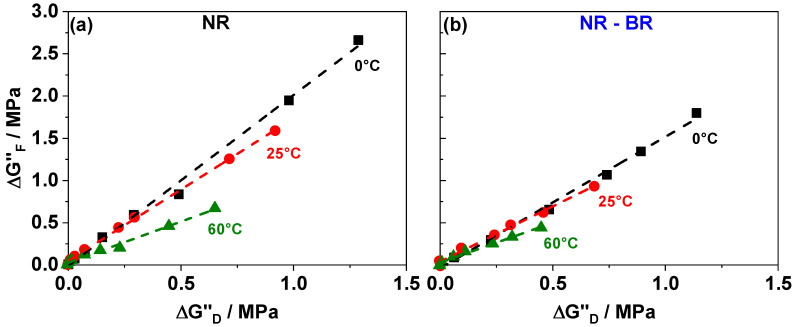
$\Delta G_{F}^{\prime \prime }$vs.$\Delta G_{D}^{\prime \prime }$ for (**a**) NR and (**b**) NR-BR composites at three temperatures 0 ${}^{\circ }$C (squares), 25 ${}^{\circ }$C (circles) and 60 ${}^{\circ }$C (triangles).

More interesting and much less investigated so far is the molecular origin of the dissipation in highly filled composites. We observed that the peak-like dissipation contributions due to fracturing of glassy bridges $\Delta G_{F}^{\prime \prime }$ as well as the sigmoidal dissipation contributions quantified by $\Delta G_{D}^{\prime \prime }$ do increase with increasing filler content (Figure 4). Plotting the values of $\Delta G_{F}^{\prime \prime }$ vs. $\Delta G_{D}^{\prime \prime }$ for highly filled samples ($\Phi_{CB}\geq \Phi_{C,CB}$) gives evidences for a linear correlation for all investigated temperatures and both rubber matrices (Figure 7). This supports the recently introduced idea [29] that both contributions, $\Delta G_{F}^{\prime \prime }$ and $\Delta G_{D}^{\prime \prime }$, are related to the filler network and that both are proportional to the initial number of glassy rubber bridges $N_{0}$. According to the proposed physical picture both dissipation contributions, $\Delta G_{F}^{\prime \prime }$ and $\Delta G_{D}^{\prime \prime }$, are related to the filler network. $\Delta G_{F}^{\prime \prime }$ is due to the heat caused by fracturing glassy bridges while $\Delta G_{D}^{\prime \prime }$ is due to the oscillatory deformation of intact glassy rubber bridges [29]. Despite of these filler network related contributions to dissipation there is a much smaller filler network independent contribution seen as $G_{\infty }^{\prime \prime }$ in strain dependent measurements. The temperature dependence of $\Delta G_{D}^{\prime \prime }$ and $\Delta G_{F}^{\prime \prime }$ can be explained within this model by the temperature dependence of the number of intact bridges $N_{0}$ or the related volume of immobilized rubber in glassy bridges. At higher temperatures the relevant number/volume is lesser due to sequential softening of immobilized rubber layers. Accordingly, the filler network related contributions to dissipation are decreasing. Considering the behavior observed at different temperatures one can conclude that the pre-factors in $\Delta G_{F}^{\prime \prime }$ and $\Delta G_{D}^{\prime \prime }$ corresponding the quantum of heat produced by breaking a glassy rubber bridge ($q_{F}$) and oscillatory deformation of intact glassy rubber bridges ($q_{D})$ show a different temperature dependence. Apart from the described similarities, Figure 7 also indicates that values of $\Delta G_{D}^{\prime \prime }$ and $\Delta G_{F}^{\prime \prime }$ are different for NR and NR-BR composites. The possible origin of these differences between both rubber composite series will be discussed in the following subsection.

Summarizing the discussion above, one can conclude that glassy rubber bridges as part of the visco-elastic filler network contribute majorly to reinforcement *and* dissipation in rubber composites. $\Delta G^{\prime }$, $\Delta G_{F}^{\prime \prime }$ and $\Delta G_{D}^{\prime \prime }$ as taken from a (modified) Kraus equation are obviously all proportional to the number of glassy bridges $N_{0}$ in corresponding rubber composites depending on filler fraction and temperature. This shows that main contributions to reinforcement and dissipation have the same molecular origin. The details are, however, influenced by the chemical composition of the glassy rubber bridges.

### 4.2. Influence of Filler Network Topology and Glassy Rubber Bridge Composition on Dissipation and Reinforcement

A main question resulting from the physical picture discussed in the last subsection is: what is truly responsible for the reported differences between NR and NR-BR based rubber composites containing identical amounts of the same filler and processed under identical conditions? From a fundamental point of view, one would expect that (i) the topology of the filler network and (ii) the chemical composition of the glassy rubber bridges could be of major importance. Both features are influenced by various factors, in particular by the chosen recipe and the processing conditions. The topology of the filler network be can changed not only by the chosen mixing procedure [34,35], but can also be influenced by the filler–matrix interaction in combination with the rubber matrix morphology [28]. In composites with two-phasic rubber matrix, the filler network topology should change if the filler in selectively incorporated in one of the rubber phases. This will not only change the filler network strength but can even influence the percolation threshold $\Phi_{c}$ since one of the phases is ‘overloaded’ with filler. The $\Phi_{c}$ value will either increase or decrease depending on whether the filler is preferentially found in a continuous or discontinuous rubber phase, respectively. The influence of the chemical composition of the glassy rubber bridges on the filler network strength (and dissipation) within the chosen physical picture is obvious since their softening behavior will depend on the type of immobilized rubber located on the interfaces. Thermodynamically the composition of the glassy bridges is controlled by rubber–filler interaction. Note that this interaction will also control the packing state of the immobilized rubber at the interfaces. Speculatively, the high $T_{g}$ values are related to a locally higher density of the rubber on filler surfaces, or in other words a significant reduction of the free volume in the glassy rubber bridges compared to the bulk state of the rubber. It is well known that small changes in the free volume fraction can have a tremendous influence on the softening behavior [36], making the extreme increase of $T_{g}$ for the immobilized rubber incorporated in the filler network better understandable.

**Figure 8:**
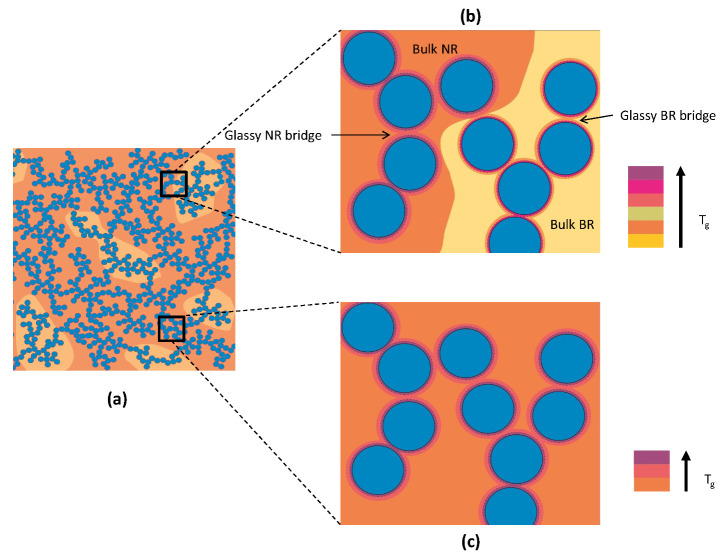
Schematic showing (**a**) viscoelastic filler network in a CB filled NR-BR blend. (**b**) Zoom on particles in filler network connected by NR and BR glassy bridges that soften several 10K higher than the bulk $T_{g}$ of respective matrix as indicated by the color code and (**c**) corresponding situation in a pure NR matrix.

Since it is found that the filler network topology is only weakly influenced in case of the carbon black filled NR and NR-BR composites investigated in this work (Figure 6), the chemical composition of the glassy rubber bridges should be the main influencing factor causing the differences between both rubber composite series. Accordingly, it is straight forward to assume that changes in strength as well as dissipation result from the NR-to-BR ratio in the glassy rubber bridges. In blend-based composites, the NR in the glassy rubber bridges is partly replaced by BR with a significantly lower glass temperature $T_{g}$. Assuming equal affinity of the filler to both rubbers, one would expect that about 30% of the glassy rubber bridges is composed of BR. However, this situation can vary, for example, due to different filler–rubber interactions. There are indications that CB prefers the BR phase in NR-BR blend composites [37,38]. Even if the properties of the glassy rubber bridges are hard to predict in detail since the reasons for the immobilization are related to the rubber-filler interaction directly at the interface, it seems to be a natural assumption that the glassy rubber bridges of the rubber with the lower bulk $T_{g}$ will also soften first during heating. The situation considered for NR-BR composites is sketched in Figure 8. A consequence of the discussed scenario is that glassy BR bridges sequentially soften first while those composed of NR start to soften at higher temperatures. This can explain why the load carrying capacity $\Delta G^{\prime }$ of the filler networks in rubber composites with NR matrix at application relevant temperatures is significantly higher compared to those in accordingly filled NR-BR composites. In the latter, the glassy BR bridges start to soften at lower temperatures accompanied by smaller volume of rubber in these glassy bridges under otherwise identical conditions. This volume argument can also explain the reduced dissipation contributions of the filler network in NR-BR composites although one has to mention that other properties, e.g., the difference between dissipation in the glassy and rubbery state, should also influence the absolute values of $\Delta G_{F}^{\prime \prime }$ and $\Delta G_{D}^{\prime \prime }$. Following these arguments one can understand the observed trends comparing rubber composites with NR and NR-BR matrix. Recently investigated silica-filled SBR-BR composites with different blend ratios show basically similar trends. However, one should note that in case of silica-filled composites, the silane groups located at the filler surfaces should also influence the overall behavior. Whether or not $T_{g}$ is always the most relevant influencing factor remains open at that point and requires further investigations. In any case, there should be additional factors influencing reinforcement and dissipation in highly filled rubber blends. However, there are many evidences showing that the discussed fundamental points—filler network topology and chemical composition of glassy rubber bridges—determine mainly the filler network related contributions to reinforcement and dissipation [28]. To validate this hypothesis based on a broader assembly of rubber composites remains a challenge, but the trends in currently available examples allowing detailed comparisons are well in line with the formulated physical picture.

## 5. Conclusions and Outlook

A comparative study focusing on the visco-elastic properties of two series of identically filled NR and NR-BR model composites has been performed. The major importance of the filler network in rubber composites containing high filler fractions for both—reinforcement and dissipation—is highlighted. It is demonstrated based on strain sweeps conducted on rubber composites with various filler fractions at different temperatures that (1) the filler network has visco-elastic properties and that (2) a modified Kraus equation promoted recently [29] is able the approximate the experimental data for the loss modulus $G^{\prime \prime }\left(\gamma \right)$ quite well. The quantification of filler network strength $\Delta G^{\prime }$ as well as filler network related dissipation contributions $\Delta G_{F}^{\prime \prime }$ and $\Delta G_{D}^{\prime \prime }$ shows that all three quantities are proportional to each other. This is understood as evidence supporting the interpretation that (3) all three major contributions to reinforcement and dissipation are related to glassy rubber bridges being part of the filler network. (4) The dissipative contributions $\Delta G_{F}^{\prime \prime }$ and $\Delta G_{D}^{\prime \prime }$ are associated with fracturing glassy rubber bridges and cyclic deformation of intact glassy rubber brides, respectively. Systematic differences in $\Delta G^{\prime }$, $\Delta G_{F}^{\prime \prime }$ and $\Delta G_{D}^{\prime \prime }$ are observed comparing NR and NR-BR composites. The values for highly filled NR-BR composites are found to be commonly 30–50% lower. (5) The influences of filler network topology and chemical composition of glassy rubber bridges for the mechanical performance are discussed. (6) An approach to quantify filler network topology and filler dispersion in highly filled rubber composites based on the size distribution of unfilled areas in TEM images is introduced. Conclusion of an analysis of TEM images for highly filled NR and NR-BR composites is that the observed differences reinforcement and dissipation are mainly due to differences in the chemical composition of the glassy rubber bridges. Speculatively, the lower $T_{g}$ values of glassy BR bridges are responsible for the observed reduction in the contributions of the filler network to reinforcement and dissipation in the compared model composites. To what extent this simplistic explanation for the observed differences between NR and NR-BR composites can applied in general is open and remains a topic for further investigations.

Future studies should focus on additional insights regarding the influence of glassy bridge composition and filler network topology on the mechanical behavior of rubber composites. This may include approaches where composition and processing conditions are varied, improvised methods providing specific information as well as numerical simulations. The common molecular origin of filler network related contributions to reinforcement and dissipation demonstrated in this work is an important insight needed to fulfill the functional requirements of rubber composites in tire treads application where dissipation and mechanical strength have to be optimized in parallel.

## Figures and Tables

**Table 1:** Fit parameters for filler network related contribution to reinforcement ($\Delta G^{\prime }$) and dissipation ($\Delta G_{D}^{\prime \prime }$, $\Delta G_{F}^{\prime \prime }$).

NR Composites									
$\Phi_{\mathrm{CB}}$	***T*****= 0**${}^{\circ }$C			***T*****= 25**${}^{\circ }$C			***T*****= 60**${}^{\circ }$C		
	$\Delta G^{\prime }$ (MPa)	$\Delta G_{D}^{\prime \prime }$ (MPa)	$\Delta G_{F}^{\prime \prime }$ (MPa)	$\Delta G^{\prime }$ (MPa)	$\Delta G_{D}^{\prime \prime }$ (MPa)	$\Delta G_{F}^{\prime \prime }$ (MPa)	$\Delta G^{\prime }$ (MPa)	$\Delta G_{D}^{\prime \prime }$ (MPa)	$\Delta G_{F}^{\prime \prime }$ (MPa)
20.4	23.33	1.29	2.66	17.28	0.92	1.59	9.48	0.65	0.67
18.9	17.60	0.98	1.95	14.42	0.72	1.25	6.80	0.45	0.46
16.0	8.87	0.49	0.83	6.38	0.29	0.57	3.32	0.23	0.20
14.0	5.91	0.29	0.59	5.33	0.22	0.44	2.70	0.14	0.17
12.3	3.49	0.15	0.33	2.20	0.07	0.18	1.77	0.07	0.12
10.0	1.83	0.07	0.16	1.25	0.03	0.11	0.90	0.01	0.06
8.5	0.95	0.03	0.07	0.87	0.01	0.06	0.50	0.00	0.03
4.4	0.22	0.00	0.01	0.17	0.00	0.00	0.09	0.00	0.00
**NR-BR Composites**									
$\Phi_{\mathrm{CB}}$	***T*****= 0**${}^{\circ }$C			***T*****= 25**${}^{\circ }$C			***T*****= 60**${}^{\circ }$C		
	$\Delta G^{\prime }$ **(MPa)**	$\Delta G_{D}^{\prime \prime }$ **(MPa)**	$\Delta G_{F}^{\prime \prime }$ **(MPa)**	$\Delta G^{\prime }$ **(MPa)**	$\Delta G_{D}^{\prime \prime }$ **(MPa)**	$\Delta G_{F}^{\prime \prime }$ **(MPa)**	$\Delta G^{\prime }$ **(MPa)**	$\Delta G_{D}^{\prime \prime }$ **(MPa)**	$\Delta G_{F}^{\prime \prime }$ **(MPa)**
20.4	17.80	1.14	1.80	11.34	0.68	0.93	6.11	0.45	0.44
18.9	13.46	0.89	1.34	7.47	0.46	0.62	4.58	0.32	0.33
17.3	10.77	0.74	1.07	5.75	0.31	0.47	3.48	0.23	0.25
15.0	6.85	0.48	0.66	4.38	0.24	0.36	2.03	0.11	0.16
12.3	3.26	0.23	0.29	2.35	0.09	0.20	1.39	0.06	0.10
8.5	1.05	0.06	0.09	0.60	0.00	0.05	0.46	0.01	0.03
4.4	0.11	0.00	0.01	0.34	0.00	−0.01	0.10	0.00	−0.01

## Data Availability

The data presented in this study are available on request from the corresponding author.
